# COVID-19 and Unfinished Mourning

**DOI:** 10.1017/S1049023X20000631

**Published:** 2020-05-12

**Authors:** Behnam Farahmandnia, Lara Hamdanieh, Hamidreza Aghababaeian

**Affiliations:** 1. School of Nursing and Midwifery, Dezful University of Medical Sciences, Dezful, Iran; 2. Department of Health in Emergencies and Disasters, School of Public Health, Tehran University of Medical Sciences, Tehran, Iran

**Keywords:** COVID-19, grief, mental health, mourning

To the Editor,

Coronavirus disease (COVID-19) is an infectious respiratory disease that first emerged in Wuhan, China in December 2019.^1^ It spread rapidly to many countries in the world, and the World Health Organization (WHO; Geneva, Switzerland) declared this virus a global pandemic on March 11, 2020.^2^ As of April 10, 2020, according to Johns Hopkins University Coronavirus Resource Center (Baltimore, Maryland USA), there were more than 1,603,330 confirmed cases in 185 countries, and at least 95,758 lost their lives.^3^ The number of confirmed cases and deaths is expected to increase in the coming days.^2^ The natural response of human beings to the death of their loved ones is expressed in grief and mourning.^4^ It is known that the traditional funeral and burial are parts of the grieving process that give mourners an opportunity to express feelings and emotions about their loved ones.^5^ Improper response to grief puts them at risk of mental health disorders (ie, depression or anxiety), persistent grief, a prolonged mourning process, as well as reduced quality of life.^6^ A study by Eleston J (2017) showed that with the outbreak of Ebola, social psychological problems were associated with increased family grief and reduced quality of life.^7^ Since COVID-19 is highly contagious, patients are dying without their families or friends by their side.^8^ As the COVID-19 pandemic evolved, large gatherings were prohibited and physical distancing was applied to contain the spread of the virus.^5^ This pandemic led to psychological crises. Lockdowns and restrictions altered the way people grieve, no manner what their culture and religion are. This limited people’s ability to mourn and restricted funeral services and rituals. The safe management of dead bodies in the context of COVID-19, that was set by governments for public health and safety reasons, led the public funeral and burial processes to change. People were deprived of the most important rituals that normally occur following a death. In the absence of these ceremonies, families and friends can’t stay in contact with the bereaved and express their support, caring, and love. Instead, they are left alone to deal with their overload of grief and emotional exhaustion. Indeed, a sense of profound sadness will remain in entire communities.^9^ It is necessary to deal with this issue with great urgency. In the current situation, mental health providers can help people to cope with grief and to strengthen them by identifying ways to move forward. This can be achieved by providing rehabilitation programs and specialized counseling to the family and relatives of the deceased, and ensuring continuous follow-up. The social and mental support can help individuals to better understand reality, organize their lives, cope with stress, and reduce the suffering caused by the loss of loved ones, to compensate the natural mourning process.
